# Hydrus Microstent implantation with OMNI Surgical System Ab interno canaloplasty for the management of open-angle glaucoma in phakic patients refractory to medical therapy

**DOI:** 10.1016/j.ajoc.2022.101749

**Published:** 2022-11-10

**Authors:** Jack Creagmile, Won I. Kim, Christian Scouarnec

**Affiliations:** Walter Reed National Military Medical Center, 8950 Brown Drive, Bethesda, MD, 20889-5600, USA

**Keywords:** Glaucoma, Hydrus microstent, OMNI Surgical system, Phakic, MIGS, Minimally invasive glaucoma surgery

## Abstract

**Purpose:**

To report a series of 8 phakic eyes of 8 patients with open angle glaucoma with uncontrolled intraocular pressure on maximum tolerable medical therapy receiving Hydrus Microstent implants combined with concomitant OMNI Surgical System ab interno canaloplasty.

**Observations:**

8 phakic eyes underwent Hydrus Microstent implantation with OMNI Surgical System ab interno canaloplasty. 2 patients underwent concurrent Kahook Dual Blade goniotomy and 1 patient underwent a concurrent micropulse transscleral cyclophotocoagulation. 6 out of 8 eyes achieved successful intraocular pressure reduction. Only 1 achieved success without the need for additional medical therapy. 1 required Neodymium-doped yttrium aluminum garnet laser to clear iris obstruction of the Hydrus inlet and 1 required selective laser trabeculoplasty for additional intraocular pressure lowering. 2 out of 8 eyes required subsequent incisional glaucoma surgery for unacceptable intraocular pressure levels despite maximum tolerable medical therapy.

**Conclusions and Importance:**

The Hydrus Microstent combined with OMNI Surgical System ab interno canaloplasty can safely and successfully reduce intraocular pressure in phakic patients with open-angle glaucoma with uncontrolled intraocular pressure on maximum tolerable medical therapy with a low complication rate and rapid visual recovery.

## 1 Introduction

The Hydrus Microstent (Ivantis, Irvine, CA, USA) is an 8mm curved device made of “nitinol” nickel-titanium alloy that is implanted into Schlemm's canal. The device has spines that dilate Schlemm's canal up to five times the native width of the canal, as well as windows that further facilitate aqueous outflow.^1^^,^^2^

The HORIZON study evaluated the effectiveness of Hydrus Microstent implantation in conjunction with cataract surgery in reducing intraocular pressure (IOP). It demonstrated that in patients with primary open angle glaucoma (POAG) and elevated IOP (off IOP lowering agents), 77% of patients achieved 20% or greater unmedicated IOP reduction out to 24 months.^3^

While results of the HORIZON trial led to FDA approval of Hydrus implantation at the time of cataract surgery, other studies have shown promise in effective IOP reduction when performed as a standalone procedure, both in phakic and pseudophakic patients. The COMPARE trial demonstrated a statistically insignificant trend towards increased IOP-lowering and drop-lowering effects of pseudophakic patients compared to phakic patients, but overall showed favorable and similar efficacy and safety profiles between both groups.^4^ Despite this, FDA approval as a standalone procedure remains off-label, and further studies are needed to further examine both the efficacy and safety profile.

The OMNI Surgical System (Sight Sciences, Inc., Menlo Park, CA, USA) is a single hand manually operated device used to facilitate the microcatheterization of the Schlemm's canal circumferentially from a single clear corneal incision. Once the microcatheter is in place the surgeon is able to perform an ab interno canaloplasty and address multiple points of outflow obstruction by delivering viscoelastic fluid to dilate Schlemm's canal and the distal outflow channels.^5^^,^^6^

The ROMEO study evaluated the effectiveness of OMNI canaloplasty and trabeculotomy in conjunction with cataract surgery in reducing IOP. It demonstrated that in patients with mild to moderate open angle glaucoma and IOP >18 mmHg, 79% of patients achieved 20% or greater IOP reduction on the same or fewer IOP lowering agents, and without needing secondary surgical intervention out to 12 months.^7^ A subsequent 2021 study showed similar efficacy in IOP reduction between phakic and pseudophakic eyes when standalone OMNI canaloplasty and trabeculotomy were performed.^8^

Both the Hydrus Microstent and the OMNI Surgical system use an ab interno approach that has shown several advantages over traditional glaucoma surgery. They are generally less invasive, spare the conjunctiva, avoid serious complications, and have a more rapid postoperative recovery. Although traditional glaucoma surgery (*ab externo* trabeculectomy and tube shunt) does provide a highly efficacious reduction in intraocular pressure, traditional surgery carries a higher risk of complications. These complications include increased rates of vision threatening complications, as well as increased rate of secondary surgical interventions when compared to minimally invasive glaucoma surgeries (MIGS) such as the OMNI and Hydrus.^9^^,^^10^

With an ever-growing arsenal of MIGS techniques and surgical devices, the exploration of finding combinations of interventions that may provide superior or synergistic IOP-lowering effects is in its relative infancy. Ab interno canaloplasty, as a standalone surgery has demonstrated good efficacy with an excellent safety profile; however, there are some concerns for how well this technique would work in patients with relatively high intraocular pressures as it dilates the canal but does not provide a bypassing of the resistance at the trabecular meshwork.^11^ Also, since there is no tensioning suture remaining in place to keep the canal of Schlemm dilated open as in ab externo canaloplasty, there is some concern that the dilatory effect on Schlemm's canal from ab interno canaloplasty would eventually fail. Hydrus microstent has the advantage of permanently stenting open Schlemm's canal and providing an inlet to allow aqueous free access to Schlemm's canal by bypassing the resistance at the trabecular meshwork, addressing the perceived weaknesses of ab interno canaloplasty. The perceived weakness of Hydrus is that it only accesses 3 clock hours of the Schlemm's canal and does not “flush” the distal outflow system with viscoelastic. Here canaloplasty may provide something theoretically advantageous by cannulating the canal 360° and flushing the distal outflow system to re-activate it if it is collapsed or atrophied. These were some of the reasons to attempt this unorthodox combination of MIGS procedures in these patients. The relative safety profile of MIGS and the lack of need to disturb conjunctiva make this an appropriate first intervention before advancing patients to a subconjunctival filtering surgery such as trabeculectomy or glaucoma drainage implants.

To date, there has been fairly little data available on this combined use of Hydrus and OMNI, but a recent 2022 case series presented at the American Society of Cataract and Refractive Surgery (ASCRS) annual meeting showed an average IOP reduction of 11.29 mmHg in a series of 16 eyes that received combined Hydrus implantation with OMNI canaloplasty.^12^ Other than this recently presented data, we are not aware of any other case series published that has evaluated the safety and efficacy of combining these two procedures.

The following case series provides early precedent for multiple potential new avenues for use of both the Hydrus Microstent and the OMNI Surgical System. Firstly, the patients in our case series represent patient populations that have previously been excluded from large cohort studies, as well as patients that meet current contraindications to Hydrus or OMNI use. These are patients with secondary open angle glaucoma (pseudoexfoliative glaucoma, pigment dispersion glaucoma), patients with juvenile open angle glaucoma, patients with severe glaucomatous disease, patients that have undergone previous trabecular meshwork altering therapies (including selective laser trabeculectomy (SLT), MIGS, and traditional glaucoma surgery), patients requiring extensive IOP lowering therapy, as well as patients with markedly elevated medicated and unmedicated IOP.^13^^,^^14^ Secondly, this case series represents off-label use of Hydrus for two reasons, both as a standalone procedure, as well as implantation in the phakic eye. Thirdly, the case series provides data on combined use of the Hydrus in conjunction with OMNI canaloplasty. We present a series of 8 phakic eyes that received Hydrus Microstents not associated with cataract surgery, following ab interno canaloplasty performed with the OMNI Surgical System. Study interventions were in patients with open-angle glaucoma and uncontrolled IOP on maximum tolerable medical therapy.

## 2 Methods

### 2.1 Surgical technique

Patients underwent canaloplasty with the OMNI Surgical System with subsequent implantation of two Hydrus Microstent implants. The canaloplasty was performed in the usual fashion as described by the manufacturer.^13^ The first of the Hydrus Microstents was placed in the nasal angle and the second was placed in the inferior angle. The ab interno canaloplasty and the nasal Hydrus implantation were performed with the surgeon sitting temporally and were both performed through the same corneal incision. The inferior Hydrus was implanted with the surgeon sitting superiorly through a second corneal incision. All cases were performed in this fashion unless otherwise specified.

### 2.2 Follow-up

All patients were seen at postoperative day 1 and postoperative week 1. At this time, follow-up was scheduled based on IOP control, medication regimen adjustments, and complications. IOP reports in the figures below represent IOP measurements on a single visit within the specified time range. If patients had multiple visits within the specified time range, each IOP measurement is reported individually.

## 3 Case series

### 3.1 Case 1

A 60-year-old Caucasian male with pseudoexfoliative glaucoma (PXG) was referred to the glaucoma service for evaluation and management. In the operative eye, the patient had pre-treatment IOP maximum of 40 mmHg, and examination revealed a cup-to-disc ratio (C/D) of 0.7 with superior rim thinning. Gonioscopy was significant for 3–4+ pigmentation of the trabecular meshwork with open angles. Humphrey visual fields (HVF) demonstrated a mean deviation (MD) of −3.00 dB, a pattern standard deviation (PSD) of 2.42 dB, and an early nasal step. Optical coherence tomography (OCT) revealed an average retinal nerve fiber layer (RNFL) thickness of 69μm with a superior thickness of 72μm and an inferior thickness of 92μm. Ganglion cell analysis (GCA) showed a global ganglion cell inner plexiform layer (GCIPL) thickness of 74μm with borderline superotemporal thinning. The patient had no history of previous glaucoma surgery or laser trabeculoplasty. Prior to the study intervention, the patient was on 6 classes of glaucoma medications (latanoprost/netarsudil, dorzolamide/timolol, brimonidine, and oral acetazolamide) with a preoperative IOP of 34 mmHg and an uncorrected visual acuity (VA) of 20/20–1. The patient underwent OMNI Surgical System ab interno canaloplasty and implantation of two Hydrus Microstents as previously described in the methodology without complication. The patient's IOP and glaucoma medication regimen in the post-operative phase are as follows (See Table 1):

**Table 1:** Post operative results for case 1.

Time	IOP (mmHg)	# of Meds
Preoperative	34	6
POD1	7	2
POW1	9	1
POM1-2	12	0
POM3-4	N/A	N/A
POM5-8	19	0
POM9-12	N/A	N/A

POD = Postoperative day, POW = Postoperative week, POM = Postoperative month.

The patient had an uncomplicated postoperative course with acceptable IOP control and an uncorrected VA of 20/20. Following postoperative month 6, the patient moved geographic locations and was discharged from the clinic at goal IOP on 0 glaucoma medications.

### 3.2 Case 2

A 37-year-old Asian male with juvenile open angle glaucoma (JOAG) was referred to the glaucoma service for evaluation and management. In the operative eye, the patient had a pre-treatment maximum IOP of 40 mmHg, and examination revealed C/D of 0.9 with inferior rim thinning. Gonioscopy was significant for a 1 clock hour patch of inferior peripheral anterior synechiae (PAS) inferiorly, as well as postoperative changes from previous glaucoma surgery (described below). HVF demonstrated an MD of −17.53 dB, a PSD of 11.73 dB, and a dense superior arcuate defect. OCT revealed an average RNFL thickness of 59μm with a superior thickness of 60μm and an inferior thickness of 57μm. GCA showed a GCIPL thickness of 64μm. The patient had a past history of trabeculectomy and iridectomy with mitomycin C, which subsequently fibrosed and failed. The patient then underwent repeat trabeculectomy with ExPRESS shunt placement which again failed due to aggressive fibrosis. There was no history of laser trabeculoplasty. Prior to the study intervention, the patient was on 2 classes of glaucoma medications (preservative free dorzolamide/timolol) with a preoperative IOP of 31 mmHg and a best corrected VA of 20/20. The conjunctiva overlying the prior two trabeculectomy sites was considered too scarred to successfully undergo bleb needling. The patient underwent OMNI Surgical System ab interno canaloplasty and implantation of two Hydrus Microstents as previously described in the methodology without complication. The patient's IOP and glaucoma medication regimen in the post-operative phase are as follows (See Table 2):

**Table 2:** Post operative results for case 2.

Time	IOP (mmHg)	# of Meds
Preoperative	31	2
POD1	15	2
POW1	12	2
POM1-2	15	3
POM3-4	16	3
POM5-8	13, 14, 12	3
POM9-12	N/A	N/A

POD = Postoperative day, POW = Postoperative week, POM = Postoperative month.

At the 1-month postoperative visit, gonioscopy showed that the Hydrus inlet had become mechanically occluded with iris tissue. Neodymium-doped yttrium aluminum garnet (Nd:YAG) laser was applied to clear the inlet of the two Hydrus microstents. At last follow up the best corrected VA was 20/20 and IOP was in the target range but requiring three drug classes: preservative free tafluprost and preservative free dorzolamide/timolol fixed combination.

### 3.3 Case 3

A 63-year-old Caucasian male with pigment dispersion glaucoma (PDG) was referred to the glaucoma service for evaluation and management. In the operative eye, the patient had a pre-treatment maximum IOP of 52 mmHg, and examination revealed C/D of 0.65 with a vertically elongated cup. Gonioscopy was significant for 2–3+ pigmentation of the trabecular meshwork. HVF demonstrated an MD of −0.05 dB and a PSD of 1.79 dB. OCT revealed an average RNFL thickness of 74μm with a superior thickness of 82μm and an inferior thickness of 104μm. GCA showed a GCIPL thickness of 62μm. The patient had a history of both argon laser trabeculoplasty (ALT) and SLT. Prior to the study intervention, the patient was on 4 classes of glaucoma medications (brimonidine/timolol, bimatoprost, and dorzolamide) with a preoperative IOP of 36 mmHg and a best corrected VA of 20/25. The patient underwent OMNI Surgical System ab interno canaloplasty with the surgeon sitting temporally. An attempt was made to implant the Hydrus microstent nasally but the distal end of the implant repeatedly re-entered the anterior chamber and would not stay in Schlemm's canal. After a few attempts, Hydrus implantation in this quadrant was abandoned and a Kahook Dual Blade (KDB) was used to perform excisional goniotomy. The Hydrus microstent was then successfully implanted in the inferior angle through a second incision with the surgeon moving to the superior position. The patient's IOP and glaucoma medication regimen in the post-operative phase are as follows (See Table 3):

**Table 3:** Post operative results for case 3.

Time	IOP (mmHg)	# of Meds
Preoperative	36	4
POD1	15	4
POW1	11	3
POM1-2	18	3
POM3-4	18	3
POM5-8	20	3
POM9-12	23	1

POD = Postoperative day, POW = Postoperative week, POM = Postoperative month.

The patient's IOP was initially controlled through postoperative month 18, at which time IOP became persistently elevated to 27–31 mmHg on multiple visits despite use of 4 classes of glaucoma medications. At this time the patient underwent Xen 45 Gel Stent implantation and has since had well controlled IOP without medications through postoperative month 1 with a best corrected VA of 20/30.

### 3.4 Case 4

A 55-year-old African American male with pre-perimetric primary open angle glaucoma (POAG) was referred to the glaucoma service for evaluation and management. In the operative eye, the patient had a maximum IOP of 30 mmHg, and examination revealed C/D of 0.6. Gonioscopy was significant for deep, open angles without significant abnormality. HVF demonstrated an MD of −2.37 dB and a PSD of 1.50 dB with nonspecific defects. OCT revealed an average RNFL thickness of 68μm with a superior thickness of 66μm and an inferior thickness of 69μm. GCA showed a GCIPL thickness of 68μm. The patient had a history of SLT in the study eye. Prior to the study intervention, the patient was on 4 classes of glaucoma medications (timolol, brinzolamide/brimonidine, tafluprost) with a preoperative IOP of 33 mmHg and a best corrected VA of 20/20–1. The patient underwent OMNI Surgical System ab interno canaloplasty and implantation of two Hydrus Microstents as previously described in the methodology without complication. The patient's IOP and glaucoma medication regimen in the post-operative phase are as follows (See Table 4):

**Table 4:** Post operative results for case 4.

Time	IOP (mmHg)	# of Meds
Preoperative	33	4
POD1	12	2
POW1	14	1
POM1-2	20	1
POM3-4	30	1
POM5-8	N/A	N/A
POM9-12	N/A	N/A

POD = Postoperative day, POW = Postoperative week, POM = Postoperative month.

The patient had acceptable IOP on minimal glaucoma drops through postoperative month 3, at which time IOP began to steeply increase and became uncontrolled on maximum tolerable medical therapy. The decision was made to perform cataract surgery in conjunction with Xen 45 Gel Stent implantation at postoperative month 4, which subsequently failed requiring Ahmed glaucoma valve (AGV) FP7 surgery an additional 5 months afterwards. The patient's IOP is now well-controlled on 2 glaucoma medications 7 months after AGV surgery with a best corrected VA of 20/20–2.

### 3.5 Case 5

A 65-year-old African American male with POAG was referred to the glaucoma service for evaluation and management. In the operative eye, the patient had a pre-treatment maximum IOP of 39 mmHg, and examination revealed C/D of 0.85 with inferior notching. Gonioscopy was significant for deep, open angles without significant abnormality. HVF demonstrated an MD of −22.14 dB, and dense superior and inferior arcuate defects. OCT revealed an average RNFL thickness of 56μm with a superior thickness of 56μm and an inferior thickness of 56μm. GCA showed a GCIPL thickness of 52μm. The patient had a past history of SLT. Prior to the study intervention, the patient was on 5 classes of glaucoma medications (netarsudil/latanoprost, timolol/brimonidine, dorzolamide) with a preoperative IOP in the 17 to 21 range with an uncorrected VA of 20/40. Because of the severity of disease, the patient was encouraged to undergo traditional glaucoma filtration surgery with either trabeculectomy or glaucoma drainage implant over the span of several years but continued to decline surgery for fear of complications. Eventually the patient conceded to undergo surgery with minimally invasive techniques to minimize the risk of complications and offer rapid visual recovery. The patient underwent OMNI Surgical System ab interno canaloplasty and implantation of two Hydrus Microstents as previously described in the methodology without complication. The patient also had micropulse transscleral cyclophotocoagulation (TSCPC) at the time of surgery. The patient's IOP and glaucoma medication regimen in the post-operative phase are as follows (See Table 5):

**Table 5:** Post operative results for case 5.

Time	IOP (mmHg)	# of Meds
Preoperative	17–21	5
POD1	11	5
POW1	14	2
POM1-2	18	1
POM3-4	15, 16	5
POM5-8	18	5
POM9-12	14	5

POD = Postoperative day, POW = Postoperative week, POM = Postoperative month.

The patient had an excellent initial response, but IOP steadily began to rise and it was noted on gonioscopy that both of the Hydrus Microstent inlets had become occluded within the iris. More medications were added, increasing up to 5 classes of drugs equivalent to the preoperative burden. From postoperative month 3–12 the IOP has been in the 14–18 mmHg range (improved from 17 to 21 mmHg range before surgery) with an uncorrected VA of 20/40-1 but is still suboptimal given the severity of visual field loss. The patient is being closely followed by the glaucoma service and has been encouraged to pursue more surgical intervention with more invasive techniques but has declined further surgical intervention at this time.

### 3.6 Case 6

A 55-year-old African American male with mixed mechanism glaucoma (POAG and iatrogenic chronic angle closure glaucoma following surgery) was referred to the glaucoma service for evaluation and management. In the operative eye, the patient had a pre-treatment maximum IOP of 40 mmHg, and examination revealed C/D of 0.8 OS. Gonioscopy was significant for an inferior patch of PAS. HVF demonstrated an MD of −4.26, with an inferior arcuate defect. OCT revealed an average RNFL thickness of 57 μm. GCA showed a GCIPL thickness of 58 μm. The patient had a past history of SLT, Xen 45 Gel Stent implantation that had failed despite bleb needling, and a subsequent failed Ahmed glaucoma valve. Prior to the study intervention, the patient was on 4 classes of glaucoma medications (brimonidine, dorzolamide/timolol, latanoprost) with a preoperative IOP of 29 mmHg and an uncorrected VA of 20/20–2. The patient underwent OMNI Surgical System ab interno canaloplasty and implantation of two Hydrus Microstents as previously described in the methodology without complication. The patient's IOP and glaucoma medication regimen in the post-operative phase are as follows (See Table 6):

**Table 6:** Post operative results for case 6.

Time	IOP (mmHg)	# of Meds
Preoperative	29	4
POD1	14	3
POW1	18	3
POM1-2	18	2
POM3-4	19	2
POM5-8	23	2
POM9-12	21	3

POD = Postoperative day, POW = Postoperative week, POM = Postoperative month.

The patient's IOP has been within the target range at all postoperative visits except postoperative month 6 when his IOP was 23 on preservative free dorzolamide/timolol fixed combination. With the addition of latanoprost the IOP was noted to be at the upper limit of the target range at 21 mmHg at the postoperative month 12 time point. At postoperative month 12 the patient's uncorrected VA was 20/15.

### 3.7 Case 7

A 65-year-old African American female with pre-perimetric POAG was referred to the glaucoma service for evaluation and management. In the operative eye, the patient had a pre-treatment maximum IOP of 30 mmHg, and examination revealed C/D of 0.75. Gonioscopy was significant for deep, open angles without significant abnormality. HVF demonstrated an MD of +1.51, a PSD of 1.89, and no glaucomatous visual field changes. OCT revealed an average RNFL thickness of 70μm with a superior thickness of 83μm and an inferior thickness of 75μm. GCA showed a GCIPL thickness of 65μm. The patient had a history of SLT. Prior to the study intervention, the patient was on 4 classes of glaucoma medications (latanoprost, dorzolamide/timolol, brimonidine) with a preoperative IOP of 29 and a best corrected VA of 20/20. The patient underwent OMNI Surgical System ab interno canaloplasty and implantation of two Hydrus Microstents as previously described in the methodology without complication. The patient's IOP and glaucoma medication regimen in the post-operative phase are as follows (See Table 7):

**Table 7:** Post operative results for case 7.

Time	IOP (mmHg)	# of Meds
Preoperative	29	4
POD1	19	4
POW1	25	4
POM1-2	21	4
POM3-4	18	4
POM5-8	N/A	N/A
POM9-12	N/A	N/A

POD = Postoperative day, POW = Postoperative week, POM = Postoperative month.

From postoperative month 1–4, the patient's IOP remained in the target range of 21 mmHg or less on 4 glaucoma medication classes. The patient was subsequently lost to follow-up until he presented at POM13 at which time IOP was found to be elevated to 26 with a best corrected VA of 20/20–1. SLT was recently performed and the patient will continue to follow closely with the glaucoma service.

### 3.8 Case 8

A 53-year-old African American male with POAG was referred to the glaucoma service for evaluation and management. In the operative eye, the patient had a pre-treatment maximum IOP of 36 mmHg, and examination revealed C/D of 0.85. Gonioscopy was significant for sparse, patchy PAS inferiorly. HVF demonstrated an MD of −4.54 dB, a PSD of 8.63 dB, and superior arcuate defect. OCT revealed an average RNFL thickness of 66μm with a superior thickness of 106μm and an inferior thickness of 48μm. GCA showed a GCIPL thickness of 68μm. The patient had a past history of trabeculectomy with ExPRESS shunt placement with additional bleb needling with mitomycin C, as well as SLT. Prior to the study intervention, the patient was on 6 classes of glaucoma medications (dorzolamide/timolol, brimonidine, netarsudil/latanoprost, oral acetazolamide) with a preoperative IOP of 23 and a best corrected VA of 20/20–1. Of note, the patient was monocular with only light perception vision in his other eye due to glaucoma. The patient underwent OMNI Surgical System ab interno canaloplasty and implantation of a single Hydrus microstent superonasally, as well as excisional KDB goniotomy inferiorly and temporally. The patient's IOP and glaucoma medication regimen in the post-operative phase are as follows (See Table 8):

**Table 8:** Post operative results for case 8.

Time	IOP (mmHg)	# of Meds
Preoperative	23	6
POD1	16	5
POW1	20	4
POM1-2	17	4
POM3-4	12	6
POM5-8	15	5
POM9-12	N/A	N/A

POD = Postoperative day, POW = Postoperative week, POM = Postoperative month.

The goal IOP is 18 mmHg or less and the IOP has been well controlled on all postoperative visits from month 1–7. Although the IOP was well controlled at postoperative month 1 on only 3 medications, the patient has elected to resume multiple glaucoma medications from before surgery to try to keep his IOP as low as possible in his only seeing eye. At postoperative month 7 the patient's best corrected VA was 20/20–1.

## 4 Discussion

### 4.1 Surgical modifications and complications

#### 4.1.1 Case 3

The surgeon experienced significant technical difficulty in inserting one of the Hydrus implants, which prompted substitution for KDB goniotomy. It should be noted that this patient had normal-appearing angles on gonioscopy and did not have any history of SLT, MIGS, or traditional glaucoma surgery.

#### 4.1.2 Case 5

The patient additionally underwent TSCPC at the time of surgery, which was planned before the time of surgery in order to maximize IOP-lowering effect in a patient with severe glaucomatous changes and highly uncontrolled IOP who was resistant to traditional glaucoma surgery.

#### 4.1.3 Case 8

The patient had a single Hydrus placed and had 2 quadrants of KDB goniotomy performed. This was performed due to the patient's prior history or trabeculectomy with ExPRESS shunt, as well as PAS identified on preoperative gonioscopy.

### 4.2 Postoperative complications and failures - POM12 and beyond

#### 4.2.1 Case 2

The Hydrus inlet became mechanically occluded with iris tissue at POM1, which was subsequently cleared with Nd:YAG laser application. There were no further episodes of occlusion and IOP was subsequently at target range.

#### 4.2.2 Case 3

The patient developed persistently elevated IOP at POM18. At this time, Xen 45 gel stent implantation was performed which has been successful in reducing IOP through the initial postoperative period.

#### 4.2.3 Case 4

The patient developed persistently elevated IOP at POM3, and Xen 45 gel stent implantation performed at POM4 subsequently fibrosed and failed thereafter. The patient then underwent AGV surgery which has been effective in controlling IOP out to POM7 from AGV.

#### 4.2.4 Case 7

After being lost to follow up from POM3 to POM12, the patient returned to the clinic at POM13, with IOP elevated to 26. SLT was subsequently performed with further follow-up and IOP response pending.

### 4.3 Limitations

#### 4.3.1 Sample size

Our study followed a relatively small cohort of patients through their course following Hydrus Microstent and OMNI canaloplasty. Although this initial data is intriguing, a larger sample size is required for a better assessment of the synergistic effect of these procedures in phakic patients.

#### 4.3.2 Heterogeneity of diagnoses

With varying diagnoses (PXG, JOAG, PDG, POAG), it is difficult to generalize treatment outcomes to a single category of glaucomatous disease. Further studies encompassing a variety of etiologies of glaucoma is needed to perform subgroup analysis.

#### 4.3.3 Effect of additional treatment

2 out of 8 patients received a postoperative laser treatment. One was a Nd:YAG laser to clear obstruction of the Hydrus by iris and the other was SLT for inadequate IOP control. It is unclear if the Nd:YAG laser procedure had a meaningful effect on the postoperative course and the SLT may have delayed the need for more incisional surgery. 2 out of 8 patients were frank failures and required additional incisional glaucoma surgery.

#### 4.3.4 Follow-up period

A longer follow up period would provide further data on the long-term outcomes of the patients and the durability of effect of the study intervention.

## 5 Conclusion

In patients with mild-to-moderate POAG, Hydrus Microstent implantation as well as OMNI canaloplasty have previously been shown to have significant and clinically meaningful reduction in IOP. However, Hydrus implantation has only been approved for use at the time of cataract surgery, and there is relatively little data to show the safety of efficacy when Hydrus and OMNI are used in combination with one another.

This case series demonstrates a novel use of combined Hydrus implantation and OMNI canaloplasty in a heterogenous group of phakic patients with uncontrolled IOP on maximum tolerable medical therapy.

Based on their reported adverse events and recovery period, both Hydrus implantation and OMNI canaloplasty are generally regarded as safe and well tolerated MIGS procedures. The visual recovery period is typically quite rapid, and serious surgical complications related to both surgeries are rare.^3^^,^^15^ In this case series of phakic patients on maximum tolerable medical therapy with uncontrolled IOP, a majority of patients saw a significant and clinically meaningful reduction in IOP even when performed as a standalone surgery. This was observed in our case study both in patients who had never had glaucoma surgery, as well those who had previously undergone both traditional filtering surgery and previous MIGS (see Table 9). However, it should be noted that very few patients in the case series were able to decrease the number of IOP-lowering agents used to achieve target IOP. In the setting of patients with uncontrolled IOP already on maximum tolerable medical therapy, standalone Hydrus Microstent implantation with OMNI canaloplasty may not be an effective means of reducing medication burden.

**Table 9:** Case summary through POM12.

	Case 1	Case 2	Case 3	Case 4	Case 5	Case 6	Case 7	Case 8
**Diagnosis**	**PXG**	**JOAG**	**PDG**	**POAG**	**POAG**	**POAG + CACG**	**POAG**	**POAG**
**Procedure**	**OMNI canaloplasty 2 Hydrus Microstents**	**OMNI canaloplasty 2 Hydrus Microstents**	**OMNI canaloplasty** **KDB goniotomy** **1 Hydrus Microstent**	**OMNI canaloplasty 2 Hydrus Microstents**	**OMNI canaloplasty** **2 Hydrus Microstents TSCPC**	**OMNI canaloplasty** **2 Hydrus Microstents**	**OMNI canaloplasty** **2 Hydrus Microstents**	**OMNI Canaloplasty** **1 Hydrus Microstent KDB goniotomy**
**Pre- operative IOP**	**34**	**31**	**36**	**36**	**21**	**29**	**30**	**37**
**Pre- operative Visual Acuity**	**20/20**–**1**	**20/20**	**20/25**	**20/20**–**1**	**20/40**	**20/20**–**2**	**20/20**	**20/20**–**1**
**Initial Number of Medications**	**6**	**2**	**4**	**5**	**5**	**4**	**4**	**6**
**Final IOP**	**19**	**12**	**23**	*failure at POM4*	**14**	**21**	**18**	**16**
**Final Visual Acuity**	**20/20**	**20/20**	**20/30**	**20/20**–**2**	**20/40**–**1**	**20/15**	**20/20**–**1**	**20/20**–**1**
**Final Number of Medications**	**0**	**3**	**4**	**2**	**5**	**3**	**4**	**5**
**Additional Procedures Performed**	**NA**	**Nd:YAG**	**Xen 45 Gel Stent**	**Cataract Extraction Xen 45 Gel Stent AGV**	**NA**	**NA**	**SLT**	**NA**

PXG: Pseudoexfoliative Glaucoma, JOAG: Juvenile Open Angle Glaucoma, PDG: Pigment Dispersion Glaucoma, POAG: Primary Open Angle Glaucoma, CACG: Chronic Angle Closure Glaucoma, KDB: Kahook Dual Blade, TSCPC: Transscleral Cyclophotocoagulation, Nd:YAG: Neodymium-doped yttrium aluminum garnet, AGV: Ahmed Glaucoma Valve, SLT: Selective Laser Trabeculoplasty.

In patients that did not achieve adequate IOP response despite Hydrus Microstent implantation with OMNI canaloplasty, it is important to note that the study intervention did not preclude or hinder further surgical or medical treatment. In this case series, both typical filtration surgery and additional MIGS were performed following Hydrus Microstent implantation with OMNI canaloplasty without event. In these patients, surgical options remained unrestricted and surgical intervention itself was not made more technically difficult due to previous Hydrus Microstent implantation and OMNI canaloplasty. In addition, patients’ IOP response to subsequent surgery was not diminished. When all these factors are considered as a whole, this case series suggests that Hydrus Microstent implantation combined with ab interno canaloplasty with the OMNI Surgical System is a reasonably safe and well-tolerated method of reducing IOP in phakic patients with open angle glaucoma with uncontrolled intraocular pressure on maximum medical therapy. Furthermore, this intervention does not preclude any further surgical treatment with standard filtration surgery or MIGS.

## Institutional review board approval

Institutional Review Board approval was obtained.

## Patient Consent

Consent to publish the case report was not obtained. This report does not contain any personal information that could lead to the identification of the patient.

## Acknowledgement and disclosures

No funding or grant support.

## Authorship

All authors attest that they meet the current ICMJE criteria for Authorship.

## Declaration of competing interest

The following authors have no financial disclosures: J.C.; W.K.; C.S.
